# Two New Anæsthetics

**Published:** 1880-06

**Authors:** J. C. Reeve

**Affiliations:** Dayton, Ohio


### Selections.

TWO NEW ANÆSTHETICS. Read before the Montgomery County Medical Society. By J. C. REEVE, M.D., Dayton, Ohio.

The dissatisfaction of the profession with the two great anæthsthetics, chloroform and ether, has long been manifested. This feeling has its origin in the dangers of the one, which seem to be more manifest with every year, and the practical disadvantages of the other, and has led to constant investigation of new compounds or trials of mixtures of the two. Bichloride of methylene is the only new agent which has attained any sound reputation, and this has scarcely been used at all, except by a single individual; and of the various mixtures of chloroform and ether, some of them used here and there to considerable extent, it may be said that no one of them has succeeded in gaining such an amount of confidence as to make its use general.

At present two new articles are rapidly coming before the profession. The claim is made for each that it is more pleasant and manageable than ether, less dangerous than chloroform. It is my purpose to-day to call your attention to these two agents, and present briefly the testimony in their favor.

The first is the bichloride of ethidene. This is a liquid, scarcely to be distinguished from chloroform in appearance or smell, with a boiling point of 58° C. and a vapor weight of 3.428. It is isomeric in chemical constitution with the dichloride of ethene, a substance which is very dangerous for inhalation, the formula of each being $C_2\ H_4\ Cl_2$. Ethidene, as it is called, is receiving attention in England, the result of the labors of a committee appointed by the British Medical Association for the investigation of anæsthetics, partial reports of which body are published in the

*British Medical Journal* of January 4th and 25th, and June 21st, 1879. The origin of the committee will be everywhere received as a guarantee of the character of the work done, and it is needless to say that the most thorough care and scrupulous exactness are shown. The experiments upon animals were by opening the trachea and administering the vapors of different anæsthetics through a tube, the arterial pressure being measured by the sphygmograph, and direct observation made of the heart's action, the sterum having been removed. Under ethidene the arterial pressure was somewhat diminished, but not nearly so much as by chloroform, and never to total extinction; nor were there the wide variations at different times in the same animal that chloroform caused. In frogs and rabbits the anæsthetic condition was produced in from four to five minutes, and kept up from twenty-six to forty minutes, the heart beating steadily all the time. In dogs anæsthesia was produced in from two to three minutes, without failure of the heart action. A marked and immediate effect upon cardiac action was produced by substituting chloroform vapor for the ethidene.

Clinical experience with ethidene has not yet been extensive. It was used already by Snow, and about three pages of his work on anæsthetics (1858) are devoted to it under the name of "monochlorated chloride of ethyl." The difficulties of manufacturing it and obtaining it pure prevented him from continuing his investigations; but he expresses the opinion, based on its chemical qualities, that it is pretty certain that it would not be liable to cause the sudden deaths which have occasionally been produced by the administration of chloroform, even if it were given freely and with no great care. He reports sixteen cases in which he administered it for various operations. In one the operation lasted ten minutes, and another must have been of longer duration, as it was for ununited fracture of the tibia and fibula. In all it acted well, and no vomiting followed, although in three cases a meal had been taken just before the inhalation. How much more information upon this anæsthetic we should have had from the lamented Snow it is impossible to say. While writing the sentence which gives the above facts, he was seized with the paralysis, which was a prominent symptom of his last brief illness.

There is a report of six administrations in the *Medical Times and Gazette*, of January 18th, 1879, by Dr. Thomas Bird, of Manchester.* Two of the patients were children, one aged three months, and the other two years and nine months. The longest time of administration was sixteen minutes. It seems to have acted kindly in all, but sickness followed in one case.

The report of the committee above alluded to gives six administrations to patients, and states that it had no injurious effect upon respiration, no indication of asphyxia were shown, and the heart's action was unaffected, anæsthesia was promptly produced, was complete, and slight retching followed in two cases. The committee indorses the opinion of Steffin, as given in Binz's Therapeutics, that, as compared with chloroform, ethidene is pleasanter, more rapid in action, produces no excitement during, nor vomiting after administration, that there is a more rapid recovery from it, and, altogether less danger attends its use.

I have not been able to obtain a specimen of this article, or I should have had some practical observations to record. Its price will, however, prove a serious bar to its introduction. The importers quoted it to me at three dollars per ounce.

Since this article was written I have had an opportunity of consulting Kappeler's new work on "Anæsthetics,"† and find that his very favorable notice of this anæsthetic closes with the following discouraging paragraph :

"Unfortunately the occurrence of a death from this article in Berlin, destroys early the hopes which had been cherished of it and will not permit of a more extended trial."

No particulars are given.

The second new anæsthetic is bromide of ethyl, or hydrobromic ether ($C_2 H_5 Br.$) It is a volatile, colorless liquid of pleasant ethereal odor, spec. gr. 1,419, thus almost as high as chloroform. It boils at 106° F. Its vapor density is 3.734; and an important characteristic of the vapor is that it is not inflammable, thus the dangers arising from administering ether by candlelight are obviated. It is needless to say that this is a very important point.

* Quoted from Turnbull on "Artificial Anæsthesia," Second Edition, 1879.

† Anæsthetica. Lieferung xx of Bilroth's *Deutsche Chirurgie*, 1880.

For the introduction of this article into practice we are indebted to Dr. Lawrence Turnbull, of Philadelphia, one of the surgeons to Jefferson Medical College Hospital; and in his work on "Artificial Anæsthesia" (sec. ed. 1879) will be found a full account and description of it.

Experiments upon animals with this liquid are lacking. A number are detailed in Dr. Turnbull's work, but they are not such as are to be desired, or as are demanded in a question of the comparative merits or the safety of a new anæsthetic. Merely to state that frogs and dogs were anæsthetized, the amount of the vapor used and the time they were under its influence, is not enough, and we very much need some careful and accurate experiments and observations with this remedy, such as those carried out by the British committee in investigating ethidene.

But if in experiments on animals we are behind ethidene with hydrobromic ether, our clinical experience has been greater. Dr. Turnbull details in his work the particulars of twenty-five administrations for various operations. Three of the cases were children of six, three and three years of age. We copy his conclusions:

| | *Min.* | *Sec.* |
|---|---|---|
| Longest time taken to place under anæsthetic influence... | 5 | |
| Shortest, ditto.... .................. .................. | 0 | 30 |
| Longest time under influence.............................. | 60 | |

Largest quantity consumed, eight ounces. Vomiting occurred in four cases after the administration; one of the patients had taken dinner before the operation.

Dr. Turnbull's example has been followed by others, notably by Dr. R. J. Levis, of Philadelphia. His experience was published in the *Philadelphia Medical Times*, and has been copied generally in medical journals, so that it is undoubtedly familiar to you, and I need give only the points generally. He has been so much pleased with it that he now uses it exclusively as an anæsthetic; has administered it for various operations, minor and capital; once for amputation of the thigh; forty minutes is the longest time of administration stated, and eleven drams the largest quantity used. He states that general excitement and struggling occur much less frequently than with chloroform or ether; that it does not influence the circulation, except to sometimes produce a slight increase in the rapidity of the heart's action; that it influences the respiration but little, and that nausea and vomiting appear to occur much less frequently than after the other anæsthetics. There are two prominent characteristics of the action of hydrobromic ether, rapidity of action, and rapidity of recovery from its effects. These were shown in Dr. Levis' observations. He estimates that its full effect is produced in one-third less time than that of chloroform, and recovery is even still more rapid.

The following trials of this new anæsthetic were made to test its merits, and to obtain personal experience of its effects. For the record of occurrences after loss of consciousness and for care and attention during administration, I am indebted to my friends Drs. Pilate and Conklin:

March 14th. Four hours after eating a moderate breakfast I proceeded to inhale the bromide of ethyl, in the recumbent position, from a bottle just opened, labeled "1 oz. bromide ethyl." About one-fourth of the contents was poured into an Allis' ether inhaler. The first and immediate sensations upon inhaling it were a sharp pungent impression on the air passages, a sense of warmth, rapidly extending, and exhilaration. Already with the second inspiration I felt a decided influence upon the brain and began to talk, anxious to continue speaking as long as possible, and to state my sensations. A rapid beating in the ears is a constant symptom with me in taking chloroform, and immediately precedes entire loss of consciousness. I remarked its presence now, and also its early appearance. It could not have been later than the third or possibly the fourth inspiration when I noted it, and this, as with chloroform, was the last sensation.

Upon opening my eyes I immediately collected myself, and remembered all; could talk clearly, and had no confusion of thought. I felt a slight sense of nausea and a feeling of languor. Eight minutes afterwards I got up and walked about without dizziness, and am confident I could have done so sooner. I did not attempt it sooner, because I felt that sickness would ensue if I arose. The feeling of nausea remained until I commenced eating my next meal, about forty minutes later.

Pulse at beginning 80, just after ascending stairs. Two drams administered. Symptoms began to be manifested after two respirations. Spoke of general warmth, pleasant sensations and beating in the ears. Anæsthesia produced in one minute and a quarter; in another quarter minute it was profound, as tested by knife point. Pulse during the first minute ran up nearly to 100, then fell during next minute to about 70, feeble and intermittent. Pupils unchanged, normal; no struggling or excitement; but tetanic clutching of the inhaler, so that it could be gotten away only with difficulty. The anæsthesia lasted only one minute and a half, then awakening without mental confusion. Pulse seven to eight minutes later, 64.

I was not satisfied with this experiment, particularly in regard to the irregularity and intermittence of the pulse, not a very assuring symptom in anæsthesia, and a result not agreeing with other observers. I had a suspicion from this fact and from the nausea that the specimen was not pure. The bottle bore the name of a house which is always a guaranty of the good quality of medicines; but in the early period of manufacture of a new article, it would not be surprising if perfection was not immediately attained. I therefore obtained another specimen*, and one week after the above trial again inhaled it. Being in the recumbent position, four hours after eating, one dram, by measure, was poured in Allis' inhaler. I aimed to take it slower this time, and counted the respirations aloud to mark when conscious action ceased. I immediately felt the same grateful and pervading glow of warmth all over the body; counted to the seventh inspiration; beating in the ears was again the last recognized impression.

Pulse before, 80; at the end of the first minute, 120; one and a half minute at the rate of 100; at the end of two minutes, 78. No irregularity or intermittence. Pupils unaffected. Totally unconscious in one minute. Consciousness returned in three minutes.

It was my design to push the inhalation farther this time, and to test muscular relaxation as well as to decide in regard to the irregularity of the pulse. Feeling that this had not been done, after about fifteen minutes, I took it again. Two measured drams were poured on the inhaler, and I placed it over my mouth and

* From the house of John Wyeth and Bro., Philadelphia.

and nose. The impression was much stronger on the nose and air passages, and the first inspiration made me cough. I then counted to third inspiration and was gone. Pupils the same as before, unaffected; pulse, before taking, 78; at the end of first minute, 124; one and a half minute 100; and of two minutes 78; no irregularity or intermittence. Anæsthesia in one minute. At the end of three minutes from the time of beginning, got up and walked across the room, and could have staid up. As an effort at prolonged anæsthesia this was not, therefore, a success. In eighteen minutes I was on my way driving to see a patient. I had not the slightest nausea after these two inhalations; felt, if anything, better than before, a sense of what the French call, *bien-être.*

My next trial of the agent and first attempt at administration was not satisfactory. The patient was a man aged about fifty, a wiry, muscular fellow, of the type and build likely to give troublesome symptoms with any anæsthetic. He was placed on the table for an operation for hæmorrhoids by Dr. Conklin. I had brought with me for the administration a large conical sponge, with which I constantly gave the A. C. E. mixture, and have no difficulty. Upon this I poured two drams of hydrobromic ether, and placed it over his mouth and nose. After one, long deep inspiration his face became deeply flushed, and he soon began to talk and then shout. More of the liquid was poured on the sponge; but his movements interfered with the inhalation of it with promptness; muscular rigidity then came on and was marked; respiration was very nearly if not quite stopped for a time by tetanic spasm of the chest. These symptoms were almost as bad as I have ever seen from ether, chloroform or the mixed vapors. I have seen worse muscular action and rigidity, but this would pass for as bad as generally met. During this time the ether was rapidly added until the supply was exhausted (13 drams), and sufficient relaxation was not produced to make the operation feasible. No observations could be made, of course, of the patient's pulse. He recovered consciousness quite rapidly, as compared with other anæsthetics, and suffered no unpleasant after effects.

This was not, of course, a fair trial of the remedy. The mode of administration was decidedly faulty. It is an ether and must be given as an ether; and that this is imperative is the lesson to be learned from this failure.

My personal experience with hydrobromic ether fully sustains the observations of others as to its exceeding promptness of action, and the rapidity with which recovery from its effects takes place. I can also say that it is pleasanter to inhale than chloroform, which is not very unpleasant, and infinitely pleasanter than ether.

Our experience must be much increased, of course, before judgment can be passed as to the relative merits of this anæsthetic. At present the testimony is positive as to the rapidity of action and efficiency, and decidedly in its favor as to safety.

I append the opinion of Dr. Levis, delivered as the result of his more extensive and later experience. "I have used bromide of ethyl in the surgery of two large general hospitals and in private surgical practice, under the most varied circumstances, which could be required to test the merits of an anæsthetic. In my use of it, in the most abnormal conditions of debility and shock of injury, in capital operations, through protracted periods of administration, in patients from early infancy to extreme old age, it has always proved satisfactory, and free from manifestations of danger. I express my conviction that it is practically the best anæsthetic known in the profession." *

* N. Y. Medical Record, March 27th, 1880.

